# A Painful Beginning: Early Life Surgery Produces Long-Term Behavioral Disruption in the Rat

**DOI:** 10.3389/fnbeh.2021.630889

**Published:** 2021-05-05

**Authors:** Douglas G. Ririe, James C. Eisenach, Thomas J. Martin

**Affiliations:** Pain Mechanisms Lab, Department of Anesthesiology, Wake Forest School of Medicine, Winston-Salem, NC, United States

**Keywords:** surgery, incision, pain, neonate, attention, anxiety, opioid, reward

## Abstract

Early life surgery produces peripheral nociceptive activation, inflammation, and stress. Early life nociceptive input and inflammation have been shown to produce long-term processing changes that are not restricted to the dermatome of injury. Additionally stress has shown long-term effects on anxiety, depression, learning, and maladaptive behaviors including substance abuse disorder and we hypothesized that early life surgery would have long-term effects on theses complex behaviors in later life. In this study surgery in the rat hindpaw was performed to determine if there are long-term effects on anxiety, depression, audiovisual attention, and opioid reward behaviors. Male animals received paw incision surgery and anesthesia or anesthesia alone (sham) at postnatal day 6. At 10 weeks after surgery, open field center zone entries were decreased, a measure of anxiety (*n* = 20) (*P* = 0.03) (effect size, Cohen’s *d* = 0.80). No difference was found in the tail suspension test as a measure of depression. At 16–20 weeks, attentional performance in an operant task was similar between groups at baseline and decreased with audiovisual distraction in both groups (*P* < 0.001) (effect size, η^2^ = 0.25), but distraction revealed a persistent impairment in performance in the surgery group (*n* = 8) (*P* = 0.04) (effect size, η^2^ = 0.13). Opioid reward was measured using heroin self-administration at 16–24 weeks. Heroin intake increased over time in both groups during 24-h free access (*P* < 0.001), but was greater in the surgery group (*P* = 0.045), with a significant interaction between time and treatment (*P* < 0.001) (effect size, Cohen *f* ^2^ = 0.36). These results demonstrate long-term disruptions in complex behaviors from surgical incision under anesthesia. Future studies to explore sex differences in early life surgery and the attendant peripheral neuronal input, stress, and inflammation will be valuable to understand emerging learning deficits, anxiety, attentional dysfunction, and opioid reward and their mechanisms. This will be valuable to develop optimal approaches to mitigate the long-term effects of surgery in early life.

## Introduction

Newborn children, both premature and full term, undergo numerous surgical procedures. Commonly these procedures are non-elective and range from emergent and non-emergent major abdominal surgery to procedures for intravascular access. Exposure to neonatal surgery and anesthesia has been associated with learning disabilities and increased diagnosis of attention deficit disorder (Wilder et al., 2009; Sprung et al., 2012; Wang et al., 2014; Ing et al., 2020). While findings regarding perioperative neurotoxicity are not uniform and are far from conclusive, discussion around the vulnerability of the neonatal brain to long-term effects still focuses on anesthesia with limited consideration of surgery-induced nociceptive somatosensory input, inflammation, and stress (Davidson et al., 2015; Barnes, 2019; McCann and Soriano, 2020). The administration of anesthesia to newborn and premature infants for painful procedures reduces morbidity through improving cardiovascular stability and reducing the stress response and is generally accepted (Anand et al., 1987). The importance of adequate treatment of nociceptive input does not seem to be restricted to the time of the surgical procedure and reducing the ongoing impact of nociceptive input and enhanced pain treatment into the postoperative period seems to be valuable as well (Anand and Hickey, 1992).

While concern about neurotoxicity of anesthetics in the neonatal brain exists, the antidote for the surgical barrage of central nervous system activation is anesthesia. Early life adversity and stress are known to produce long-term changes in learning and reward processing, and surgery is a stress producing exposure (Birnie et al., 2019). To mitigate stress, the standard of care for surgery is the provision of anesthesia, resulting in analgesia and loss of consciousness, or complete anesthesia using local or regional techniques. That is to say, the untreated and unrelenting noxious input, stress, and inflammation can result in altered neuronal development that may be pathologic through accelerating synaptic connectivity where it should remain limited and winnowing connections that should normally be maintained. This may result in vulnerability to altered behaviors and susceptibility to insults that exists beyond childhood extending throughout life (Ririe, 2015).

Evidence suggests that the exposure to anesthesia and surgery during critical periods of development may produce long-term changes in somatosensory processing with increased sensitivity to pain in later life (Peters et al., 2005; Hermann et al., 2006; Schmelzle-Lubiecki et al., 2007). The changes in processing are not restricted to the dermatomes of the previous surgery, implicating higher center involvement in the central nervous system. Interestingly, the impact long-term from injury early in life in the rat paw seems to be less robust and of shorter duration in younger animals with no residual behavioral effect peripherally demonstrable in the adult, although inflammatory memory may exist (Ririe et al., 2003; Ririe and Eisenach, 2006; Moriarty et al., 2019). Paw incision in particular generates abnormal peripheral activity in neurons and in the spinal cord that are substantial, but recovery is swift and the abnormal input resolves rapidly. Despite this, subtle differences may still exist into adulthood despite no gross behavioral changes (Van Den Hoogen et al., 2018).

Long-term effects of early life pain experience occur in humans as well as in animal models, and the critical period in the animal model seems to occur in the first postnatal week in the rat (Hermann et al., 2006; Walker et al., 2009). This first postnatal week in the rat is thought to correspond neurodevelopmentally from preterm to term in the human; this is a time of heightened vulnerability with the neonatal rat similar to a human premature infant and the 1 week old rat being correlated with the term human infant (Rice and Barone, 2000; Semple et al., 2013). The focus of this study is on the effects at the term, or near term, neurodevelopmental stage, recognizing that more robust long-term effects of surgery that likely occur in the central nervous system may occur earlier in development and may have less impact as development continues and neural connections become more established. This is a critical period with many changes that render the developing central nervous system more vulnerable to effects that may last a lifetime (Ririe, 2015). This is also a time when exposure to surgery is likely.

Early life stress is associated with increased risks of disrupted behaviors in later life including anxiety, substance abuse, attentional dysfunction, anxiety, and others (Novick et al., 2018). Learning dysfunction is a particular concern in children after surgery and anesthesia and reinforcement learning and maladaptive reward processing likely share a common link (Dalley and Everitt, 2009; Wilder et al., 2009). Since surgery is considered a stressful procedure, in addition to causing inflammation and pain, the long-term behavioral effects from surgery focused on both those related to stress and those related to nociceptive input and anesthesia and included measures of anxiety, depression, learning, attention and reward circuitry. In this study we hypothesized that peripheral surgery and anesthesia during early development produces long-term disruptions in these behaviors that rely on higher level brain regions for long-term phenotypic changes to better understand the neurobehavioral sequela of surgery and anesthesia in early life. The studies presented were limited to male animals due to the nature of the long-term complex behavioral studies. Sex is an important biological variable to understand and explore in this paradigm as exposure to surgery using the incision model has sex related differences in microglial responses suggesting even at birth sexually dimorphic responses exist in the rat short and long-term (Joshi et al., 2019; Moriarty et al., 2019). Additionally, sex differences exist in the behavioral outcomes for anxiety, learning, attention and opioid self-administration (Aguirre et al., 2020; Gogokhia et al., 2020; Kimbrough et al., 2020). Given the variability of the complex behavior studies and the high probability of sex related differences, exploring sex differences was considered not feasible in this initial study with the primary outcome related to surgery. This resulted in limiting studies to one sex to reduce animal number and variability for this foundational and seminal work to permit focusing on differences from exposure to surgery with anesthesia and anesthesia alone in the early life independent of sexual differences. This by no means diminishes the importance of sexual differences in exposure to surgery. On the contrary, the importance of sexual differences are critical to explore robustly and finding a significant impact of early life surgery on long-term behaviors emphasize the importance of further studies in females to fully understand the complete biological consequences of surgical exposure during critical periods of development.

## Materials and Methods

### Animals

**FIGURE 1:**
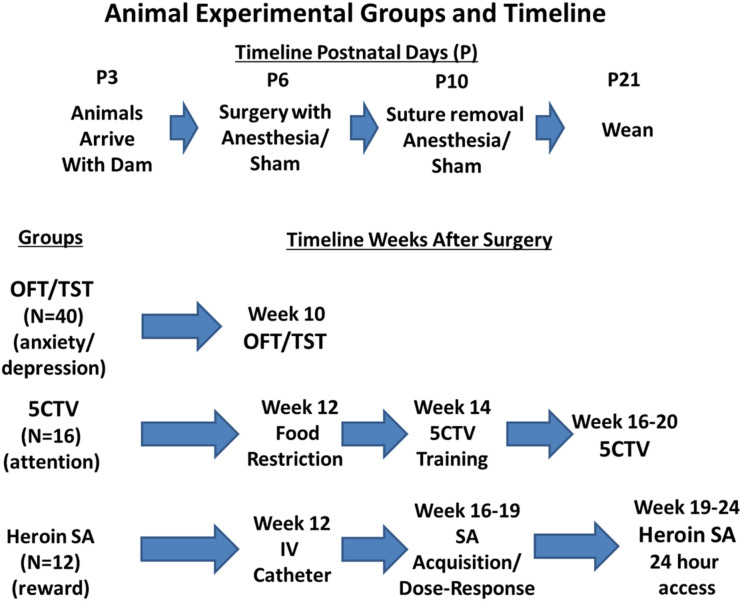
Experimental groups and behavior outcomes timeline. Animals used in the study were male Sprague Dawley pups. Random assignment was made of 80 male pups from 8 dams to either have treatment at postnatal day 6 (P6) of anesthesia and surgery or anesthesia alone with half of the pups in each group from each dam. Sutures were removed at P10 and animals weaned at P21. Random assignment of animals into behavioral outcome measurement was made with equal numbers of surgery and anesthesia and sham anesthesia from each dam; 16 animals were placed in the five choice serial reaction time titration variant (5CTV) group for measurement of attention and distraction, 12 animals were placed into the heroin self-administration (SA) group, and 40 animals were placed into the open field discrimination (OFT)/Tail Suspension Test (TST). Twelve additional animals were not included as no animal was excluded from loss or failure to train and all animals initially entered into the experiment randomly were used for the experiment. As a result a total of 68 animals were studied as only 16 could be studied in the 5CTV and 12 in the self-administration paradigm.

Sixty-eight male postnatal day (P) 6 Sprague-Dawley littermate pups were studied (Envigo, St. Louis, MO, United States) from a total of 80 P3 male animals (80 male pups and 8 dams) that arrived at our facility with dams in 3 cohorts; 40 animals for open field and tail suspension, 20 for animals audiovisual distraction in the 5 Choice Serial Reaction Time Titration Variant (5CTV), and 20 animals for heroin self-administration (Figure 1). A total of only 16 animals could be studied in the 5CTV and 12 in the self-administration paradigm. However, due to the long-term nature with complex surgical procedures and training, additional animals were kept in case of loss of animals during surgery or infection or failure to train. No animal was excluded from loss or failure to train and all animals initially entered into the experiment randomly were used for the experiment. As a result a total of 68 animals were studied as only 16 of 20 were studied in the 5CTV and 12 of 20 in the self-administration paradigm. Half the pups in each litter had surgery and anesthesia and half had anesthesia alone (sham) (same anesthesia; isoflurane 2%), allocated randomly. Animals were housed together with the dam in litters of 8–12 pups until weaning. After weaning, animals were housed in similar treatment pairs. Housing consisted of a climate-controlled room under a reverse 12-h light/dark cycle in AAALAC approved facilities. All behavioral experiments were conducted during the dark phase. The use and handling of the animals was in accordance with the guidelines provided by the National Institutes of Health and the International Society for the Study of Pain and approved by the Institutional Animal Care and Use Committee of the Wake Forest University Health Sciences.

### Incisional Surgery

All animals were separated from dam for the same time as other pups during the surgical procedure. The incision group underwent general anesthesia with isoflurane 2% under spontaneous ventilation, the dorsal surface of the paw was prepped with povidone-iodine and an incision was made and closed with 2 inverted mattress sutures as previously described (Ririe et al., 2003). The sham animals underwent the same isoflurane general anesthesia and povidone-iodine prep. Animals were awake and ambulating within 5 min after the anesthetic. Animals were given unique numbers and identified according to number for the remainder of the study. No animal in the study had a wound dehiscence or infection during the study; therefore, all animals were included in the data analysis. Animals were recovered together on a heating blanket. Animals were returned to the dam simultaneously, after all in the litter were treated. One week later the sutures were removed under brief general anesthesia with isoflurane. Sham animals were also anesthetized for a similar time with isoflurane at this time. Animals were weaned at P21. After weaning at P21 rats were housed in pairs and given free access to standard rat chow and water.

#### Anxiety and Depressive Behavior

##### Open field test

Open field (OF) testing was used as a measure of anxiety. OF exploratory behavior was assessed using commercially available equipment and software (Med Associates Inc., St. Albans, VT, United States) as previously described (Martin et al., 2004). Briefly, animals were placed in activity chambers divided into 5 equal zones, one in each corner and 1 in the center to analyze zone preference in free exploration. Activity chambers consisted of acrylic enclosures measuring 42.5 × 42.5 cm that were 37.5 cm tall with an open top. Duplicate banks of 16 infrared transmitters spaced 2.5 cm apart were placed in both the X and Y directions, 2.5 cm above the floor surface, with aligned infrared detectors on the opposing sides of the chamber. A third bank of infrared transmitters and detectors was located in the X direction, 7 cm above the floor surface such that the rats used for these studies were required to rear on their hind limbs to interrupt these beams to detect vertical counts. Each activity chamber was housed within a light- and sound-attenuating enclosure. There were no gaps or dividers in the zones. Data were collected in 6-min bins for 1 h. Measures collected included distance traveled, total beam breaks in both the X and Y direction (ambulatory counts) and vertical counts.

##### OF analysis

Forty animals were used for OF testing at 10 weeks after either surgery and anesthesia (*N* = 20) or sham anesthesia alone (*N* = 20) and placed in the apparatus one at a time. Zonal analysis was performed for center field and corners. Data were obtained by a person blinded to the treatment groups. The primary outcome measure was crosses into the center zone for anxiety. Secondary outcome measures included ambulatory counts in the center zone, time in the center zone, total distance traveled in the X-Y plane, and vertical counts total and center.

##### The tail suspension test

The tail suspension test was utilized to determine the long-term effects on a depression phenotype (Chermat et al., 1986). Briefly, adhesive tape was placed on the tail of the animal from the base of the tail all the way to the tip and the tape was attached to a hook to suspend the animal without undue force on the tail. The animal was left suspended for 5 min in a three-sided chamber with a distance of the nose to the ground being approximately 20 cm when suspended and videotaped from the open area. Animals were recorded one at a time. An observer blinded to treatment scored the time to first stop struggling and the total time struggling and immobile.

##### Tail suspension analysis

Ten weeks after the surgery, the same animals used for OF exploration (40 animals from either surgery and anesthesia (*N* = 20) or sham anesthesia) were placed one at a time in the apparatus. Immobility was defined as a complete lack of movement in the four limbs and trunk. The primary outcome measure was the total amount of time struggling over the 5-min time period. The secondary outcome was the time to first stop moving.

#### Five Choice Titration Variant Protocol and Behavior

##### Five choice titration housing and apparatus

Briefly, after weaning at P21 rats were housed in pairs and given free access to standard rat chow and water. At 12 weeks following the initial surgery, animals were singly housed and given *ad lib* access to rat chow until they attained a minimum body weight of 240 g. Animals were then reduced to 90% of their free feeding weight and given sufficient rat chow thereafter to maintain normal growth and increased weight gain while maintaining 90% of average free feeding weight for Sprague Dawley rats based on growth curves from the vendor. Animals were given free access to water except during experimental sessions. All procedures were conducted in commercially available five choice operant chambers with a stainless steel grid bar and sound and light attenuated boxes and controlled through a computer interface using Med-PC IV software (Med Associates Inc., St. Albans, VT) as previously described (Martin et al., 2015). At the top of the wall with the food trough a standard stimulus lamp with a red lens cap (house light) and an adjustable sonalert tone generator were placed (Med Associates Inc.).

##### 5 choice titration variant training and behavior

**FIGURE 2:**
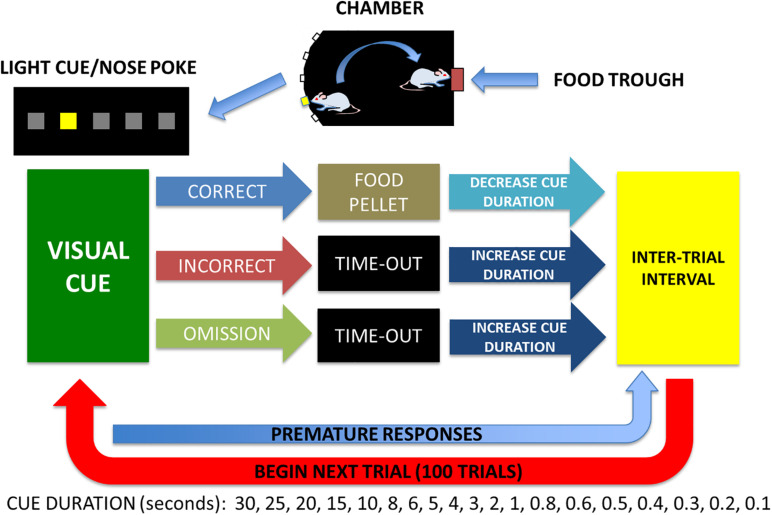
Five choice titration variant to measure attentional performance. A light and sound attenuated activity chamber is used with a bank of 5 lights on one wall and a food trough with a light on the opposite wall. Animals are trained in stages to: (1) poke head in trough for food when lighted, (2) poke head in center cue light and get food reward in trough, (3) poke head in any cue randomly lighted and get food reward in trough, and (4) poke head in any cue randomly lighted of varied duration (the titration) of illumination based on performance. Median cue duration (MCD) was calculated from trials 25–75 and used for assessment of attention on day 1 for baseline and an audio and visual distraction was performed from trials 25–75 on days 2, 3, 4 and no distraction on day 5. Premature responses are nose pokes any time during the inter-trial interval and only reset the inter-trial interval and are not considered a trial and do not contribute to the MCD.

All experiments were conducted during the dark phase of the light:dark cycle and once body weight stabilized at 90% of free feeding weight as previously described (Martin et al., 2015). A simplified diagram of the 5CTV training and procedure is presented (Figure 2). In phase 1, animals were trained to nose poke in the food trough in response to a light and reinforced by a 45 mg chocolate flavored rat chow pellet (Bio-Serv Inc., Flemington, NJ, United States) accompanied by a 0.5 s tone and turning off the food trough lamp for 0.5 s. Sessions lasted for 30 min or until the animal obtained 100 pellets, whichever occurred first. Once animals obtained 100 pellets for a minimum of 2 consecutive sessions they moved to phase 2.

In phase 2, the animals were trained to nose poke in the middle of the 5 nose poke holes located on the wall opposite of the food trough for food pellet reward as previously described (Martin et al., 2015). Briefly, each trial consisted of the light in the middle nose poke being illuminated for 30 s [30 s cue duration (CD)]. A nose poke resulted in the light being turned off and the food trough light being illuminated with delivery of two food pellets. Head entry detection at the food trough initiated a 2 s reward cycle timer, after which the food trough lamp was turned off and an inter-trial interval (ITI) timer of 5 s was initiated. Incorrect or failure to poke the middle nose poke within 30 s [limited hold (LH)] resulted in the light being turned off and a 2 s time-out period with all lights off. Responses in any of the nose pokes during this time-out period reset the 2 s time-out timer. At the end of the time-out, the next trial was initiated signaled by illumination of the house light and after the ITI, illumination of the middle nose poke light. Sessions consisted of 50 trials or 30 min, whichever came first. Animals were required to complete all 50 trials with a minimum of 80% correct responses for 3 consecutive sessions and then proceeded to phase 3. In phase 3 any one of the five nose pokes was illuminated randomly and all else was similar to phase 2. Animals needed to complete all 50 trials with a minimum of 80% correct responses and then moved to the final titration phase.

In phase 4, the titration variant paradigm of the five choice serial reaction time task (5CTV) was introduced and training was identical to the third phase of training, except that the CD and LH were altered based upon the outcome of each trial as previously described^6^. Each session consisted of 100 trials or 30 min whichever came first. The CD array series was 30, 25, 20, 15, 10, 8, 6, 4, 2, 1, 0.8, 0.7, 0.6, 0.5, 0.4, 0.3, 0.2, and 0.1 s. The LH was set to the CD or 5 s, whichever was greater. The CD was initially set to 30 s. If the animal made a correct response, the CD was decreased in series for the next trial. If the animal made an incorrect response or an omission, the CD was increased in series for the next trial. If the animal made an incorrect response or omission when the CD was 30 s, or made a correct response when the CD was 0.1 s, the CD was not altered for the subsequent trial. The median cue duration (MCD) was calculated from trial 16–100 (excluding the first 15 trials during which animals were titrating down the CD). Once the MCD was stable and titrated to < 1 s duration for a minimum of 5 consecutive sessions (100 trials per session and the MCD calculated from trials 16–100), the animal was considered fully trained.

##### Experimental manipulation audiovisual distraction (dstr) protocol

A white stimulus lamp (Med Associates Inc) was placed in the top center of the chamber as described previously (Martin et al., 2015; Ririe et al., 2017). Briefly, the lamp was illuminated at approximately 3 Hz (alternating 0.16 s ON, 0.16 s OFF) during trials 25–75 of individual sessions. The tone generator (2.9 kHz) was utilized to simultaneously create an audio distraction of 89 decibels and was synchronized with the light distraction stimulus and delivered during the same trial period. Effects of DSTR on performance in the 5CTV were determined from trials 25–75 of three separate sessions on 5 separate days: PRE: baseline session day 0 with no DSTR, DSTR: during the audiovisual distraction session day 1, 2, 3, and POST: session on day 4 with no DSTR. All parameters for performance in the 5CTV were determined during the 25–75 trials over the 5 days and compared. Sixteen animals were entered into 5CTV behavior, 8 surgery and anesthesia and 8 sham anesthesia. All animals entered completed training and were used for analysis. The primary outcome measure related to attention was the MCD that was calculated using Microsoft Excel from the cue durations for trials 25–75 for each session (the time of the distraction). Secondary outcome measures were number of correct, incorrect, omission, and premature responses as well as latency to emit correct responses, incorrect responses, or to retrieve the food reward during the same time period.

#### Heroin Self-Administration Protocol and Behavior

##### Heroin self-administration

Rats were implanted with chronic indwelling catheters for intravenous administration of drugs as described previously (Martin et al., 1996, 1998). Briefly, rats were anesthetized with pentobarbital (50 mg/kg, i.p., Abbott Laboratories, North Chicago, IL, United States) after pretreatment with atropine methyl nitrate (10 mg/kg, i.p., Sigma, St Louis, MO, United States) and penicillin G procaine (75,000 U, i.m., Wyeth Laboratories, Philadelphia, PA, United States) and implanted with venous catheters placed in the right jugular vein. The catheter was guided into the right jugular vein until it terminated just outside the right atrium and anchored to muscle in the area of the vein. The other end of the catheter continued subcutaneously to the back where it exited between the scapulae through an implanted polyethylene shoulder harness encased in Teflon mesh. The harness provided a point of attachment for the catheter to a needle-tubing leash that passed out the top of the animal chamber. A leak proof swivel attached the leash to the tubing leading to the infusion pump to permit almost complete freedom of movement. The rats were allowed to recover for 7 days before the initiation of experimental procedures. After recovery from surgery, animals were trained to self-administer intravenous infusions of 0.06 mg/kg heroin during daily 4 h sessions on a fixed-ratio 10 schedule of reinforcement (Martin et al., 1996). Heroin hydrochloride was obtained from the Drug Supply Program of the National Institute on Drug Abuse. Experimental chambers contained a house light, a stimulus light, response lever, and a tone source. The operant chambers were housed in sound- and light-attenuating cubicles and daily self-administration sessions were conducted 5 days/week. Each drug infusion was paired with a 20-s tone and light stimulus and the response lever was retracted during the TO periods and at the end of the session.

##### Self-administration experiments

Twelve rats (6 surgery and anesthesia and 6 anesthesia sham) were entered into the study. All animals entered completed training and were used in analysis. Once drug intake was stable (1 mg⋅kg^–1^⋅d^–1^), a dose response curve was determined over 3 sessions followed by access to self-administration 24 h/d (Martin et al., 1996). The schedule for increasing heroin doses, determined from the rate of drug self-administration until the final infusion rate (6 mg/kg) produced intakes of up to 366 mg⋅kg^–1^⋅d^–1^, is presented in Results. The rats were maintained on this schedule of increasing heroin doses for 21–30 days. The primary outcome was heroin-self administration during 24-h access. Secondary outcomes were acquisition of heroin self-administration and dose response differences.

#### Data Analysis/Statistics

Primary outcomes for each study were identified prior to examining data. Sample size was determined based on the primary outcome for the OF and suspension test and were based on OF crosses to the center zone with a sample size of *N* = 20 in each group needed to provide 80% power assuming a two-sided α = 0.05 and to detect a difference of 100 crosses and standard deviation of 90. Two other behavioral cohorts, the 5CTV and the heroin self-administration were exploratory in nature and therefore no power calculation was performed and the maximum number of animals that could be studied on one cohort was selected (16 5CTV and 12 heroin self-administration with equal numbers of surgery and sham for each).

Prior to analysis, parametric assumptions were evaluated for all variables using histograms and descriptive statistics. Data are reported as medians (range) if not normally distributed or means (standard deviation) if normally distributed. For histogram analysis of groups data means are presented as means (with standard error of the mean). Student’s *t*-test and repeated measures analysis of variance (ANOVA) were used for normally distributed data, and Friedman test and Mann Whitney *U*-test were used for not normally distributed data. Fisher’s exact test was used for comparison of group training in the 5CTV. *Post hoc* analyses were conducted using Holm-Sidak correction for multiple comparisons across time after surgery for MCD distraction data for the 5CTV using baseline data prior to surgery as control. Significant differences in variances between groups for 24-h heroin data resulted in using a linear mixed model for statistical inference to reduce type 1 error using 2-way ANOVA. Random intercepts were utilized to account for the repeated measures (i.e., over time) within animals. Each model was assessed for best fit with the addition of random slopes (i.e., varying slopes across subjects) using likelihood ratio tests, but no model fit was improved so fixed slopes were employed. Briefly the model was optimized and fixed effects analysis of deviance was determined using type II Wald F tests with Kenward-Rogers df to generate p-values. Animal was used as a random effects component and the effect of animal on variance determined. Statistical tests were performed using R version 3.3.2 and Systat Software Inc., San Jose, CA. Corrections for multiple comparisons were used where appropriate. There are no missing data or outliers to report and no animals were excluded from the study. Where *P*-values are reported, these are corrected *P*-values. *A priori* only corrected *P* < 0.05 was considered statistically significant.

## Results

There were a total of 68 male animals studied, 34 animals that underwent surgery and anesthesia and 34 sham animals that underwent anesthesia alone at postnatal day 6 (P6). Each dam was a foster and had half of the litter with surgery and anesthesia and half sham anesthesia control. The duration of general anesthesia averaged 6 ± 1 min and was not different between incision and sham animals [*t*(1,66) = 0.573; *P* = 0.570]. The average duration of separation from the mother was 30 ± 8 min and was no different between incision and sham groups [*t*(1,66) = 0.427; *P* = 0.676]. Seven days later, the surgery and sham animals underwent a brief 2 ± 1 min general anesthetic for suture removal and there was no difference between groups [*t*(1,66) = 0; *P* = 1].

### Anxiety and Depressive Effects of Early Life Incision

**FIGURE 3:**
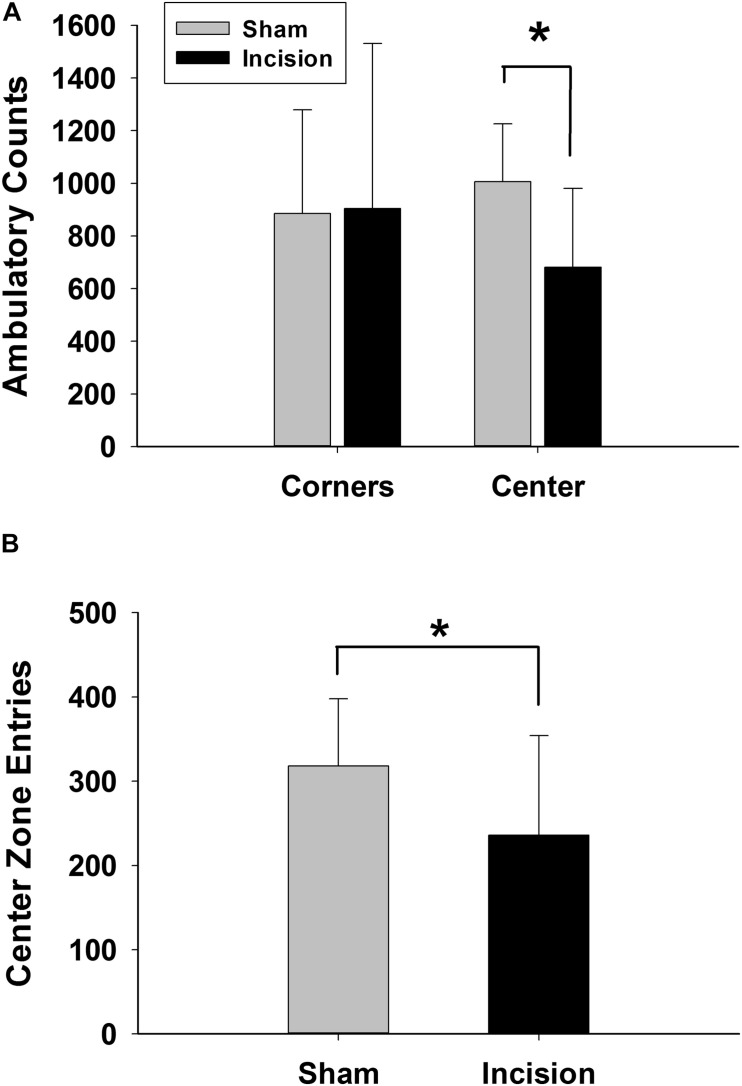
Anxiety measured with open field exploration. Open field testing was performed at 10 weeks after incision and anesthesia (*N* = 20) or anesthesia alone (sham) (*N* = 20). **(A)** Ambulation in the center zone is reduced in the incision group while there is no difference in ambulation in the corners between the two groups representing reduced exploration of the center zone once entered. **(B)** Center zone entries are significantly reduced in the incision group which suggests increased anxiety and reduced exploration in the open zone. *Statistically significant difference between groups (unpaired *t*-test).

Open field discrimination was used as a measure of anxiety. The primary outcome in the open field was zonal analysis and revealed a decrease of entries into the center zone in the incision group (*N* = 20) compared to the sham (*N* = 20) (318 ± 80 entries sham and 237 ± 118 entries incision group [*t*(1,38) = 2.549; *P* = 0.03] (Figure 3). Secondary outcomes in the open field analysis showed a decrease in ambulatory counts in the center zone for the incision group (1005 ± 220 counts for sham and 681 ± 300 counts for the incision group [*t*(1,38) = 3.888; *P* < 0.001] (Figure 3), decrease total distance traveled in the incision group compared to the sham group (8080 ± 2012 for the sham and 6631 ± 2290 for the incision group [*t*(1,38) = 2.125; *P* = 0.04] and decreased time in the center zone for the incision group compared to the sham group (4.7 ± 1.2 min in the sham versus 2.0 ± 1.6 min in the incision group [*t*(1,38) = 4.079; *P* < 0.001]. No difference between groups in total vertical counts (sham 178 ± 92 and incision 156 ± 63; *t*(1,38) = 0.886; *P* = 0.381) or vertical counts in the center zone were present (sham 25 ± 14 and incision 18 ± 12; *t*(1,38) = 1.602; *P* = 0.117).

**FIGURE 4:**
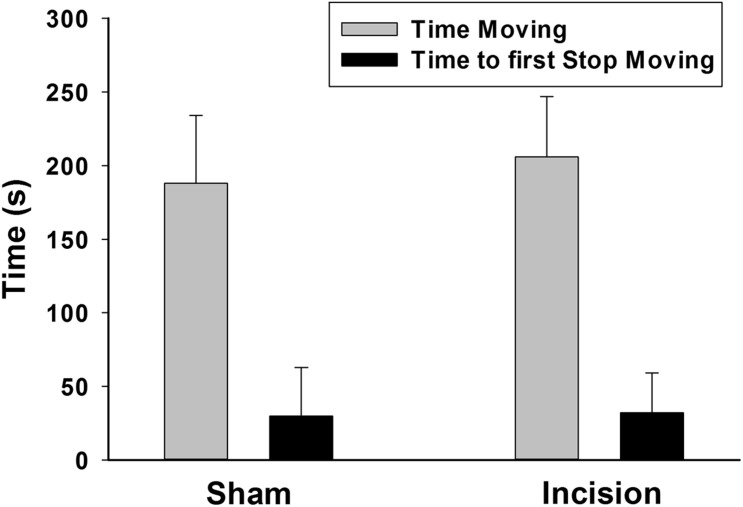
Depression or despair measured in the tail suspension test. The tail suspension test was conducted at 12 weeks after in the initial incision. There was no difference found between the anesthesia and incision (*N* = 20) and the anesthesia alone (sham) groups (*N* = 20) with respect to the time to first stop moving and the total time moving during the test (unpaired *t*-test).

The tail suspension test was used as a marker of depression or despair. No difference was found in the primary outcome of total time struggling between groups (*N* = 20 each group) (206 ± 41 s for the sham and 188 ± 46 for the incision group [*t*(1,38) = 1.299; *P* = 0.202] (Figure 4). The secondary outcome of time to first stop struggling was not different (32 ± 27 s for sham compared of 30 ± 33 s for the incision group [*t*(1,38) = 0.273; *P* = 0.787].

### Behavioral Effects of Early Life Incision in the 5CTV

Sixteen animals of the 68 were randomly enrolled in the 5CTV study and all made it through the stages of training (8 animals in the incision group and 8 animals in the control group). Training was slower in the animals that underwent incision. This was found to be isolated to phase 2 training and was apparent as soon as task difficulty began to increase in progressing from nose poke in the food trough for food pellets in phase 1 to nose poke the single center light and then getting pellets in food trough on the opposite wall. When all 8/8 (100%) of sham animals successfully completed phase 2 training, only 3/8 (38%) of the incision animals had completed phase 2 [Fisher’s exact (*P* = 0.03)], although all animals successfully completed phase 2 [median time to complete phase 2 control 5 days (range 5–7) versus 7 days (range 5–12)].

**FIGURE 5:**
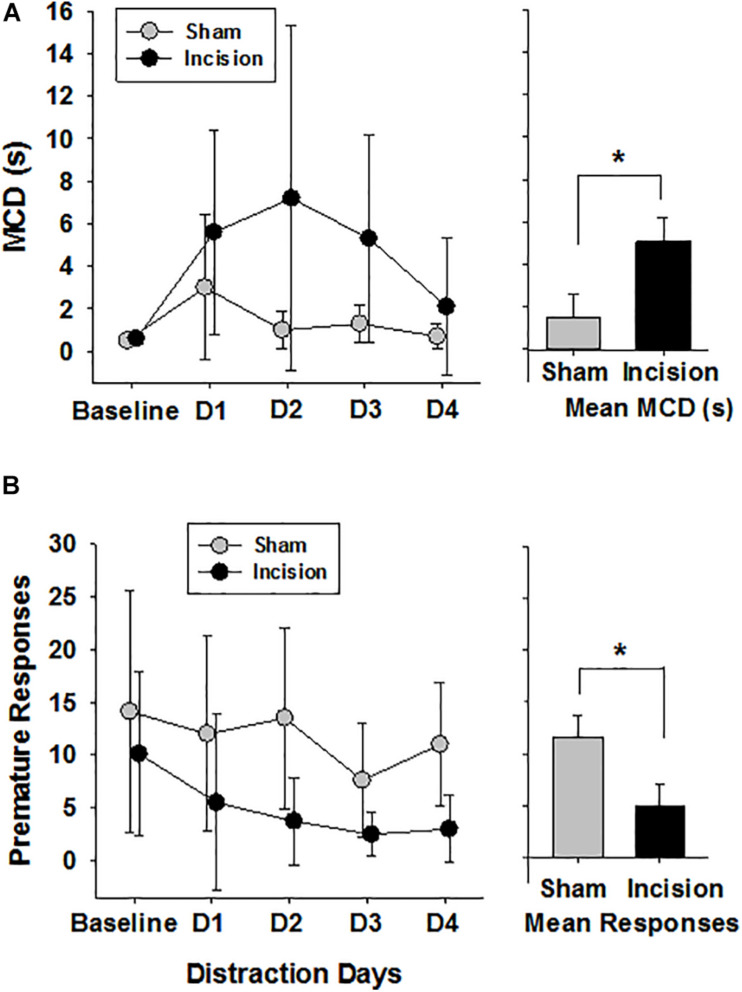
Attention and Distraction after Early Life Surgery. Attentional performance was assessed using the median cue duration (MCD) from trials 25–75 at baseline and for 3 days of audiovisual distraction and then no distraction on Day 4 (*N* = 8 anesthesia alone (sham) and *N* = 8 incision and anesthesia animals). **(A)** No difference in baseline MCD between sham and incision was present (unpaired *t*-test). Distraction over days impaired performance in animals as measured by an increase in the MCD in both groups of animals (two way repeated measures ANOVA). In animals after early surgery, the five choice serial titration variant showed a persistent disruption seen as elevated MCD during days 1 to 4 (D1–D4) while exposed to distracting light and noise during task compared to control anesthesia sham. The incision group MCD was increased compared to baseline at all time points and remained elevated from baseline on Day 4 with no distraction (Holms-Sidak pairwise correction for comparisons). The bar graph shows a significant difference between group means. **(B)** Premature responses in the five choice serial titration variant were determined reflecting decreased inhibitory control when increased and either more inhibitory control or increased anxiety when decreased. Baseline responses were no different (*N* = 8 control anesthesia alone and *N* = 8 incision and sham anesthesia animals) (unpaired *t*-test). There was an effect of distraction and a treatment effect with only the animals in the surgery group with reduced premature responding from baseline with distraction and not the sham group. The bar graph shows a significant difference between group means. Data are presented as means and standard deviation and bar graphs are group means and standard error of the mean. *p < 0.05 between groups.

At baseline in the 5CTV the MCD was no different between the incision and the sham groups (0.5 ± 0.1 s in the sham and 0.6 ± 0.2 in the incision group [*t*(1,14) = 0.594; *P* = 0.56]. The effect of surgery on distraction in the 5CTV is presented (Figure 5A). There was overall an interaction between treatment and the effects of distraction on MCD over time [*F*(4,56) = 3.28; *P* = 0.018]. A significant effect of distraction across days on the MCD in both groups was present [*F*(4,56) = 17.96; *P* < 0.001], and there was also a significant treatment effect with a difference between the incision group and the sham [*F*(1,56) = 4.89; *P* = 0.044]. The peak effect of distraction in the incision group was on the second day of distraction and the mean MCD was 9 × greater than the mean MCD in the control group on the same day. Pairwise comparison revealed a difference in MCD from baseline on the first day of distraction only for the sham l group (day 2), but days 1, 2, and 3 of distraction were all different from baseline and day 5 for the incision group (*P* < 0.05) reflecting an inability to acclimate to the distraction in the incision group. Additionally on day 5, after the three days of distraction, the MCD remained different from baseline in the surgery group (0.6 ± 0.2 at baseline and 2.1 ± 3.2 on day 5 after distraction in the incision group (*t* = 2.75; *P* = 0.032).

**FIGURE 6:**
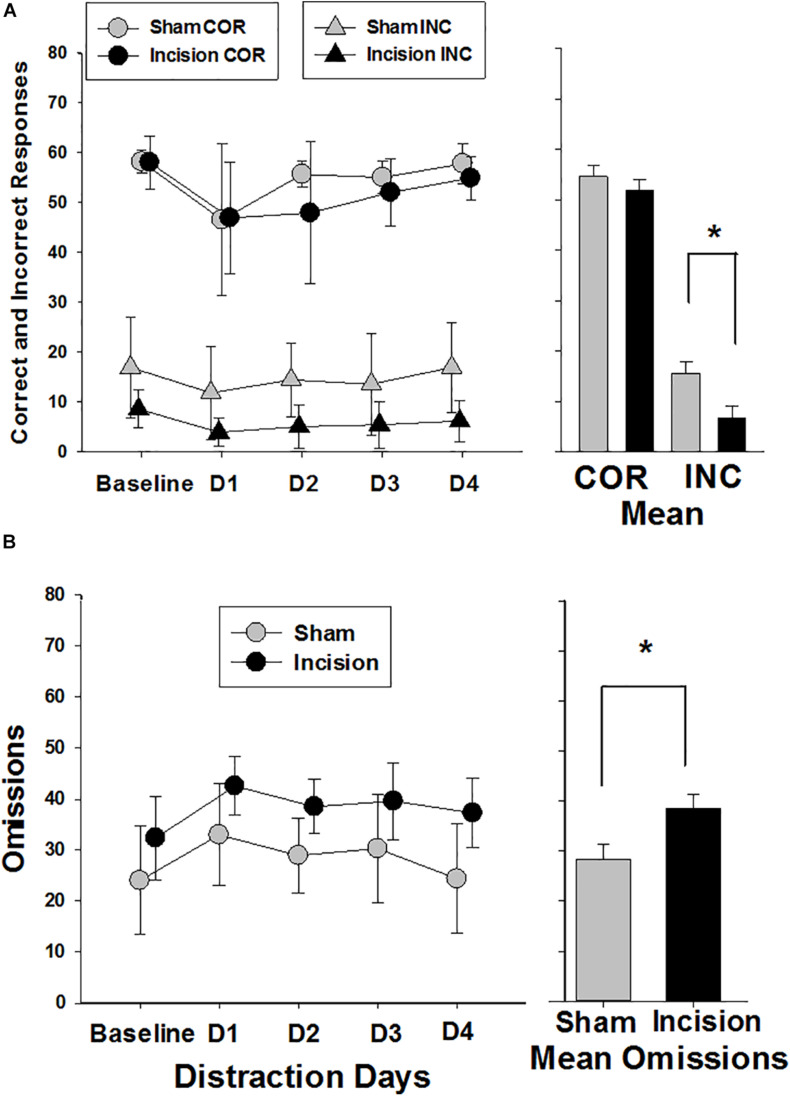
Correct, Incorrect and Omission Responses during the attention trials. Responses are shown for sham (*N* = 8) and incision and anesthesia animals (*N* = 8). **(A)** Correct responses are shown at the top of the graph (circles). No difference in baseline correct response between sham and incision was present (unpaired *t*-test). Distraction decreased correct responses in both incision and sham with no difference between groups (two way repeated measures ANOVA). The bar graph shows no significant difference between correct group means. Incorrect responses are shown at the bottom of the graph (triangles). No difference in baseline incorrect response between sham and incision was present (unpaired *t*-test). Distraction decreased incorrect responses in both incision and sham with a significant difference between groups (two way repeated measures ANOVA). The bar graph shows a significant difference between incorrect group means. **(B)** Response omissions are shown with no difference in baseline omission responses between sham and incision (unpaired *t*-test). Distraction increased omissions in both incision and sham with an overall difference between groups (two way repeated measures ANOVA). The bar graph shows a significant difference between omission group means. Data are presented as means and standard deviation and bar graphs are group means and standard error of the mean. *p < 0.05 between groups.

Correct responses were similar in both groups at baseline (58.1 ± 2.2 and 58.0 ± 5.3; *t*(1,14) = 0.045; *P* = 0.962) and there was an effect of distraction over time in both groups [*F*(4,56) = 7.85; *P* < 0.001] with reduced number of correct response with distraction and no treatment effect between groups [*F*(1,56) = 0.765; *P* = 0.397] (Figure 6A). Incorrect responses were lower in the incision group compared to the sham at baseline, but did not reach significance [9.4 ± 3.9 and 17.8 ± 10.1; *t*(1,14) = 2.19; *P* = 0.056)]. With distraction the incorrect responses decreased in both groups significantly [*F*(4,56) = 5.832; *P* < 0.001] with a greater decrease in incorrect responses in the incision group compared to the sham [*F*(1,56) = 7.305; *P* < 0.001] and no interaction with respect to the number of days of distraction [*F*(4,56) = 0.564; *P* = 0.690]. Omissions were not different at baseline between the two groups (24.1 ± 10.8 and 32.6 ± 8.4, sham and incision, respectively, [*t*(1,14) = 1.76; *P* = 0.10] and omissions increased in both groups with distraction over time [*F*(4,56) = 13.601; *P* < 0.001] with a significant treatment difference [*F*(1,56) = 5.697; *P* = 0.032] and no interaction [*F*(4,56) = 0.737; *P* = 0.571] (Figure 6B). Premature responses were not different between the two groups at baseline (14.1 ± 11.4 and 10.1 ± 7.8, sham and incision, respectively, [*t*(1,14) = 0.819; *P* = 0.426)] and there was a distraction time [*F*(4,56) = 4.776; *P* = 0.002] and treatment effect [*F*(1,56) = 5.697; *P* = 0.032] with no interaction [*F*(4,56) = 0.916; *P* = 0.461]. This represents a significant decrease in premature responses with distraction over time in the incision group [*F*(4,28) = 3.332; *P* = 0.024] and not the sham group [*F*(4,28) = 2.347; *P* = 0.079] (Figure 5B).

Latency to correct, incorrect and reward were all no different at baseline between the two groups (correct: 1.2 ± 0.9 and 1.1 ± 0.5 s, sham and incision, respectively [*t*(1,14) = 0.275; *P* = 0.788]; incorrect: 1.5 ± 0.4 and 1.6 ± 0.7 s, sham and incision, respectively [*t*(1,14) = 0.351; *P* = 0.731]; and reward: 1.4 ± 0.2 and 1.2 ± 0.2 s, sham and incision, respectively [*t*(1,14) = 2.00; *P* = 0.065)]. All latencies increased with distraction over time [correct: *F*(4,56) = 7.576; *P* < 0.001, incorrect: *F*(4,56) = 3.099; *P* = 0.022, and reward: *F*(4,56) = 10.371; *P* < 0.001]. There were no treatment effects for latencies [correct: *F*(1,56) = 0.906; *P* = 0.357, incorrect: *F*(1,56) = 0.121; *P* = 0.733, and reward: *F*(1,56) = 0.074; *P* = 0.789] and no significant interaction [correct: *F*(4,56) = 1.294; *P* = 0.283, incorrect: *F*(4,56) = 0.099; *P* = 0.982, and reward: *F*(4,56) = 0.493; *P* = 0.741].

### Effects of Early Life Incision on Heroin Self-Administration

**FIGURE 7:**
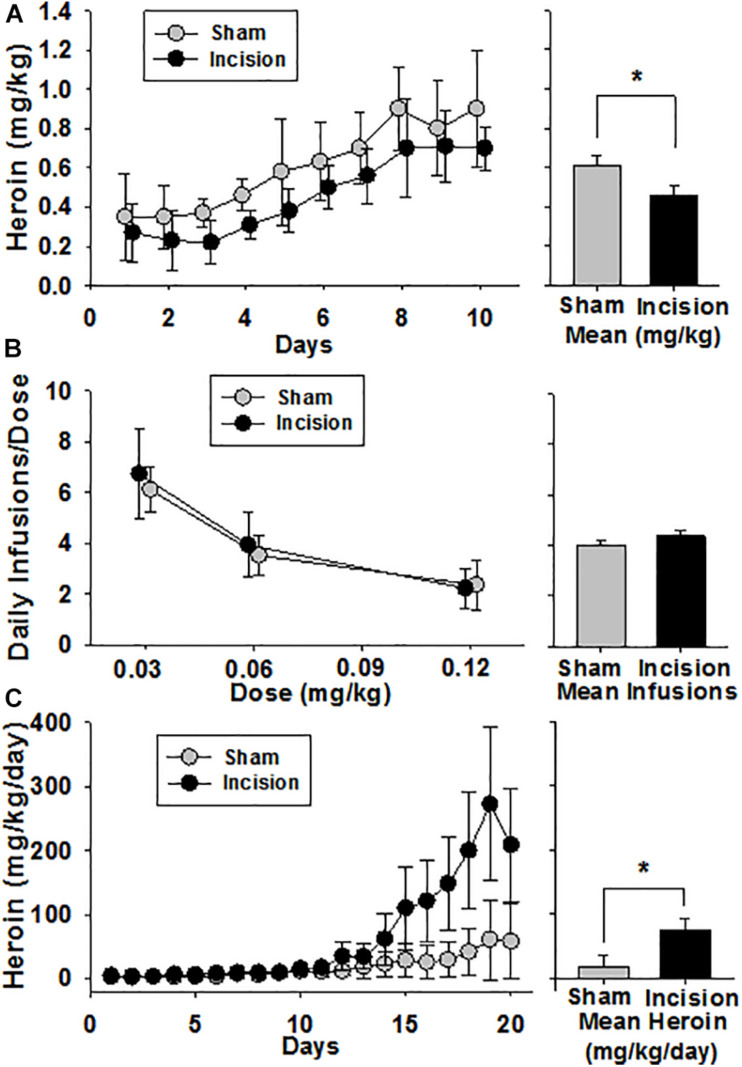
Heroin Self Administration after Early Life Surgery. Heroin self-administration was initiated at 12–16 weeks after surgery and anesthesia (*N* = 6) or the anesthesia alone (sham) (*N* = 6). **(A)** Acquisition was reduced in the incision and anesthesia group compared to sham. No difference was seen at baseline or after 10 days at steady state between the 2 groups. The bar graph shows a significant difference between group means. **(B)** After steady-state heroin self-administration within session was achieved, a dose response was performed over 3 sessions which revealed a dose-dependent decrease in heroin consumption in both groups, but no difference between groups. The bar graph shows no significant difference between group means. **(C)** Animals were given 24-h access to heroin. No difference between baseline heroin consumption was present. There was escalation of heroin intake in both groups compared to baseline over time. Animals after early life surgery self-administered/escalated heroin more than sham (mixed model with random effects). The bar graph shows a significant difference between group means. Data are presented as means and standard deviation and bar graphs are group means and standard error of the mean. *Statistically significant difference between groups.

Twelve animals from the same dam randomly underwent surgery under anesthesia (incision) (*N* = 6) or anesthesia alone (sham) (*N* = 6). Heroin self-administration acquisition occurred over a period of 10 days. Baseline amount of heroin self-administration on day one was no different between groups (0.27 ± 0.15 mg/kg in the incision and 0.35 ± 0.22 mg/kg in the sham group [*t*(1,10) = 0.792; *P* = 0.447)]. There was an increase in heroin self-administration over time [*F*(9,90) = 28.871; *P* < 0.001] and also a group effect on the acquisition of self-administration over the ten days with the incision group taking heroin more slowly [*F*(1,90) = 5.106; *P* = 0.047] and no interaction between the two [*F*(9,90) = 0.353; *P* = 0.954] (Figure 7A). On day 10 the amount of heroin intake was no different (0.70 ± 0.11 mg/kg in the incision group and 0.89 ± 0.30 mg/kg in the sham group [*t*(1,10) = 1.51; *P* = 0.162]. This level did not change over the next seven days.

A dose response was then performed over 3 sessions. There was a dose-dependent decrease in heroin infusions with increasing heroin dose of 0.03, 0.06, and 0.12 mg/kg/dose in 3 hour sessions [*F*(2,35) = 29.739, *P* < 0.001] (Figure 7B). However, no group effect in the dose response was seen [*F*(1,35) = 1.416, *P* = 0.262] and no interaction was found [*F*(2,35) = 0.199, *P* = 0.821]. Following the determination of the dose response curve, the animals were given 24 h access to 0.06 mg/kg/infusion of heroin for 21 days. Due to wide variances between groups and to limit type I error using a 2 way ANOVA, a linear mixed model was used to test for effects using time and treatment as fixed effects and random effects were repeated measure within an animal over time with fixed slope. There was an increase in heroin self-administration over time in both groups with greater heroin intake in the incision group compared to sham (Figure 7C). The fixed effects analysis revealed a treatment effect from incision compared to sham as a neonate which revealed that the treatment effect was responsible for 56.1 mg (95% CI: 32.5–79.6) [*F*(1,10) = 5.2; *P* = 0.045] difference in heroin consumption over the study. A time effect was determined to be 2.7 mg (95% CI: 1.9–3.5) [*F*(1,226) = 120.6; *P* < 0.001] of heroin increase for each 24-h interval. There was also a significant interaction between time and treatment which yielded a 10.8 mg (95% CI: 9.6–12.0) [*F*(1,226) = 53.5; *P* < 0.001] of heroin difference between groups. This represents a large effect of incision on heroin consumption (effect size, Cohen *f* ^2^ = 0.36). The random effects structure that was fit revealed a substantial interaction of the animal response to the variance in the model random intercept with the animal contributing 27.7% (95% CI: 25.7–29.7) to the total variance of the model.

## Discussion

This study focused on the behavioral consequences of surgical incision and anesthesia in early life and demonstrated maladaptive behaviors in adult animals. The most striking findings are the constellation of increased anxiety in the incision group manifest as reduced exploratory behavior, reduced premature responses in the attention task (5CTV), and delayed acquisition of self-administration of heroin. Learning is altered with impairment in the surgery group which manifests as longer training in the 5CTV and slower acquisition of heroin self-administration, with some behavioral changes inter-related. Interestingly, despite slower acquisition of heroin self-administration, when given 24-h access to heroin, greater escalation of heroin intake occurs in the surgery group suggesting that alterations in the reward circuitry may partially underlie these maladaptive behaviors.

**FIGURE 8:**
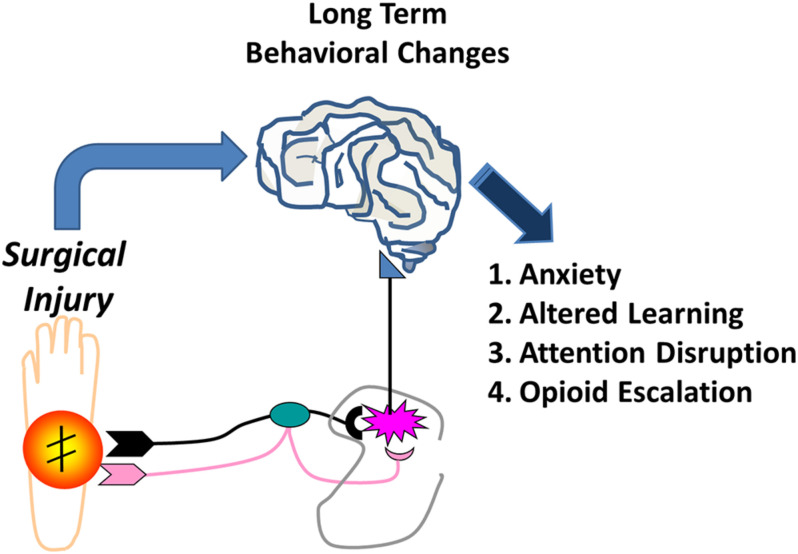
Early Life Surgery Behavioral Effects. Effects of surgery in early life result in long-term behavioral changes in anxiety, learning, attention, and altered opioid reward behavior. Early life surgery and anesthesia likely produce these effects through a systemic response or neural pathway from the stress, inflammation or noxious nociceptive activation. Transient or permanent changes in the brain during development are likely responsible for long-term changes that result in altered or maladaptive behaviors in later life.

Anxiety, measured by reduced exploration of the center zone in the open field in our study, suggests that neonatal surgery acts as an early life stress that results in anxiety (Lähdepuro et al., 2019; Juruena et al., 2020). Stress in early life results from a myriad of events from maternal separation, to birth trauma, to surgery. The effects of stress from surgery acutely are well studied, but long-term effects in the adult far removed from the surgery itself are interesting. Certainly a barrage of nociceptive input during circuit development is stressful and as such contributes to enhancing other aspects of stress production. Early life stress has been associated with psychopathologic illnesses, including both anxiety and depression, depending on type of stress, timing, duration, or underlying genetic and epigenetic factors (Lowry et al., 2008; Palmisano and Pandey, 2017). As such it is better to conceptualize stress as altering basic aspects of affective and cognitive function that manifest in different ways (Novick et al., 2018). Although animals in this study were separated from the mother, both groups were separated for the same amount of time. Thus, it is unlikely that maternal separation by itself is responsible. Regardless stress may be one aspect of the impact of surgery in the brain and long-term effects (Figure 8), although there is certainly overlap between stress and nociceptive input.

Disordered HPA axis is associated with affective disorders and animals bred for anxiety have altered glucocorticoid responses and after early life stress have impaired glucocorticoid regulatory feedback in the brain and anxiety (McIlwrick et al., 2016; Ke et al., 2018). Surgery and pain stimulate the HPA axis. Early life surgery may alter the HPA axis and alter stress responsiveness and contribute to long-term changes in glucocorticoid effects, possibly in the amygdala, that manifest as anxiety (Anand and Aynsley-Green, 1988; DeKeyser et al., 2000; Cheng et al., 2019). Studies to further delineate the age dependent differences in HPA responses to surgery and pain as well as the lasting impact on adult stress responses will be valuable in understanding the emergence of anxiety and altered responses to stress in the adult.

The concept that early life events may alter development of reward circuitry is not novel and pain has been implicated (Matthews et al., 1999; Matthews and Robbins, 2003; Novick et al., 2018; Birnie et al., 2019). Delayed acquisition of heroin self-administration coupled with accelerated intake when given free access seems to highlight an underlying impairment in reward circuitry. One thought is that learning is a reward related function and that addiction may be related to dysregulated reward circuitry and learning deficits and that learning is directly dependent on the intact reward pathways (Hyman et al., 2006). Consistent with altered opioid acquisition, acquisition of cocaine self-administration is altered with maternal separation, although the etiology may be different than for opioids (Matthews et al., 1999). Data suggest long-term changes in somatosensory function occur from surgery and this could contribute to long-term changes in the brain. In neuropathic pain one can find altered opioid responsiveness commonly manifest as reduced rewarding effects of opioid, possibly related to reduced opioid receptors in the ventral tegmental area (Ozaki et al., 2004). As such early life surgery and altered somatosensory processing could contribute to escalation of heroin intake.

Attention is important for optimal performance of tasks relying on cognitive and executive processes and the rat is no different. We used the novel 5CTV to assess attention in adult rats after the early life surgery since previous studies in humans have suggested attention deficits are more likely after early life surgery and anesthesia (Sprung et al., 2012; Ing et al., 2020). The animals in this study could perform the attentional task under normal conditions with a maximal performance that was no different, but after early life incision, animals developed an inability to accommodate to a distraction. This is demonstrated by the increase in MCD that did not return to baseline. The altered attention, likely altered by increasing attentional load with distraction, disrupts information processing and this results in decreased correct responses, increased omissions and decreased incorrect responses as the MCD increases. Additionally because of the titration component, there is more balance between correct responses and omissions or incorrect responses via the up-down method that is quite different from the classical 5 choice paradigm (Martin et al., 2015). In the incision group, premature responses also are reduced after distraction and could reflect activation of inhibitory circuits as the use of distraction seems to engage activation of the prefrontal cortex (Ririe et al., 2017, 2018). The persistent distraction could just be another facet of impaired learning in that the incision animals may not be able to “learn” that the audiovisual distraction is not a threat. Also motivation could be a factor, but the baseline motivation was not different. In the incision group, premature responses are reduced with distraction. This is different than in the classic 5 choice where premature responses are increased with distraction (Carli et al., 1983). The reason for this difference is unclear. Increased premature responses in the classic 5 choice may result from disorientation or non-specific arousal and this disorientation could contribute to delayed decision to engage in the 5CTV and result in the reduced premature responses. Activation of inhibitory circuits is possible as distraction seems to engage activation of specific brain areas, but likely the reduced premature responses is another reflection of reduced willingness to engage (Ririe et al., 2017, 2018). More likely, however, the persistent dysfunction that occurs and the decrease in premature responses are related to attention deficits whereby the early life surgery impairs the ability to engage in the attention task as the attention is persistently redirected toward the distraction. Curiously, while attention deficit and anxiety are different, they commonly occur together. We have found both anxiety and attentional dysfunction as a result of the early life incision. Whether they occur in the same animals to the same extent or not will require future studies to evaluate the linkage between the behaviors.

One explanation for the heroin self-administration is that reward circuitry is blunted resulting in slower acquisition of self-administration, learning to self-administer. The change in the paradigm is that when given free access, the impaired reward response requires higher doses of heroin to achieve the same rewarding effect which is manifest in the free access paradigm with self-administering greater overall amounts of drug to achieve a similar reward. This may also result from altered circuitry or from more rapid development of tolerance to opioid rewarding effects related to early life pain and endogenous opioid effects at the time of the initial surgery. Alternatively, impaired emotional regulation has been associated with increased opioid intake and this may go along with the anxiety phenotype (Garland et al., 2017). This impaired emotional regulation is associated with early life adversity and propensity to maladaptive reward related behaviors (Duffy et al., 2018). This does not necessarily imply anhedonia, *per se*, but may suggest blunting of normal response to reward. This would require greater stimulation to achieve the same neurochemical response and is consistent with underlying mood dysregulation, anxiety, playing a role in reduced exploration of rewarding stimuli (Green et al., 2019). Consistent with this, in early life stress, dopamine signaling is disrupted and is associated with increased anxiety behavior and alcohol intake and this may occur after early life surgery resulting in blunted reward leading to escalation of heroin (Karkhanis et al., 2016).

While investigation has focused on early life stress, studies on effects of pain at critical developmental periods on long-term maladaptive behavior are relatively sparse. Our data of elevated opioid intake from neonatal surgery are consistent with data using rats with neonatal visceral pain (Norwood et al., 2014). In other models, early life pain in rats results in increased ethanol preference in adults similar to early life stress (Anand et al., 1999; Roman and Nylander, 2005; Karkhanis et al., 2016). Increased anxiety in the adult and reduced food intake in a novel environment are also associated with early life pain (Anand et al., 1999; Bhutta et al., 2001; Low and Fitzgerald, 2012). These are consistent with our findings of slower training in the food-reinforced 5CTV and slower acquisition of heroin self-administration, although reward/motivation or anxiety may both play a role.

Stress and pain are certainly related (Victoria et al., 2013, 2014, 2015; Henderson et al., 2015; Victoria and Murphy, 2016a, b; Averitt et al., 2019; Harder and Murphy, 2019). Other than the HPA axis, a number of underlying mechanisms for the effects of early life stress have been postulated including microglial effects in the brain, corticotropin regulation and signaling, and epigenetic alterations in dopamine receptor expression, among others (Sasagawa et al., 2017; Birnie et al., 2019; Catale et al., 2020). The findings that there is possibly a memory of the early life pain from injury in glia suggest altered regulation of the central nervous system responses are possible in the future, certainly in the spinal cord, but this may occur in the brain (Beggs et al., 2012; Ririe, 2015). This could occur directly via peripheral neural input or through a systemic activation of inflammation (Figure 8). This may predispose to vulnerability to further abnormalities in processing or inflammatory responses in the central nervous system throughout life related to injury, infection, or pathologic input.

A few limitations are present in this study. Most notable is that the surgery was only done in animals at a single age and sex. Additionally, the role of the nociceptive input, and the role of extent of injury, and finally the effects of longer duration procedures and therefore longer duration anesthetic have not been addressed. In this study, we were interested in the effect of surgery. As such anesthesia alone/sham was the control group and no anesthesia/no surgery control was not studied. This is a limitation as anesthesia alone has profound effects on long-term behavior, but this is largely reported for longer duration exposures and single exposures of an hour or less have been associated with little long-term behavioral changes in outcomes ranging from memory to attention (Liu et al., 2014; Qiu et al., 2016; Xiao et al., 2016; Huang et al., 2017; Murphy et al., 2017). Though the effects of the anesthetic alone were not evaluated in this study, this data on short exposure is consistent with the short anesthetic alone in our study not resulting in any behavioral changes that we have been able to appreciate thus far. Nevertheless, future studies will need to include the no anesthesia/no surgery control group. Lack of this group does not change the results presented whereby surgery produces outcomes long-term that are different than anesthesia alone, but the effect of anesthesia alone could not be determined since no normal control animals (no anesthesia/no surgery) were included. Further studies to better delineate the combined effect of anesthesia and surgery will require this group. Another limitation in this study is that no analgesia was provided after the initial surgery and emergence from the general isoflurane anesthetic. Analgesics may modify the development of maladaptive behaviors (Victoria et al., 2015). However, the optimal interventions and timing are also not fully appreciated. Opioids are not currently regarded positively as newborn exposure may contribute to abnormalities in cognitive and motor development (Kesavan, 2015). The data on opioids early in development are inconclusive. It will be valuable to understand the role of opioids for analgesia on maladaptive behaviors as to whether they alter learning and self-administration of opioids and the role of pain at the time of exposure. Along these lines, while an opioid-free perioperative period has been suggested to be beneficial, this may or may not mitigate effects of surgery and stress during critical periods of development. While the opioid controversy is inconclusive with data on both sides, the dominant practice today is to reduce narcotic use in the newborn period. The effect of narcotic in the early life period is unclear with regard to opioid requirements in later life for pain and in the predisposition to opioid use and abuse. Additionally, the effects of other modalities of pain control including reducing inflammation with non-steroidal or steroidal anti-inflammatory drugs, opioids, and other modes of analgesia in mitigating or exacerbating the effects will be valuable. While the incision surgery is very limited, examination of larger and more invasive surgical experiences will be valuable to understand the roll of the peripheral extremity injury better and surgical impact more completely. In this light, further investigation of the role of opioids and other adjuvants during surgery will be valuable to understanding long-term effects on learning and reward and to design rationale approaches to surgical perioperative care of neonates and infants for the best short- and long-term outcomes.

We have demonstrated altered behaviors long after an early life surgical incision and anesthetic in male animals. These behaviors are controlled in the brain and therefore, while understanding the role of analgesics will be valuable, clearly establishing the roles of the different responses to surgery on regional brain activity will be essential. Moreover, these findings make it imperative to establish the impact of sex differences on early life exposure to surgery on the outcomes in the adult. Establishing and defining the vulnerable period and brain regions should also be insightful. Finally, it seems that the story of brain and behavioral effects is complex and establishing the role and contribution of the components of surgery that lead to maladaptation will be important; in this case studies on inflammation, nociceptive input, and stress will help our understanding of the underlying mechanisms and approaches to therapy (McCann and Soriano, 2020). These studies will help put into context our findings, enable hypothesis-generated studies to reduce the negative impact of early life surgery and pain, and develop translational studies to determine the human correlates.

## Data Availability Statement

The raw data supporting the conclusions of this article will be made available by the authors, without undue reservation.

## Ethics Statement

The animal study was reviewed and approved by Wake Forest University School of Medicine Animal Care and Use Committee.

## Author Contributions

DR, TM, and JE designed the research and drafted the manuscript, which all authors edited. DR and TM performed the experiments and analyzed the data. All authors contributed to the article and approved the submitted version.

## Conflict of Interest

The authors declare that the research was conducted in the absence of any commercial or financial relationships that could be construed as a potential conflict of interest.
